# Detection of Enteroviruses on Environmental Surfaces in Daycare Centers Using Droplet Digital PCR (ddPCR) and Its Public Health Implications

**DOI:** 10.3390/pathogens15020161

**Published:** 2026-02-02

**Authors:** Kyung-Seon Kim, Hye-Jin Jang, Seo-Youn Koo, Jeong-Hyun Lee, In-Hae Choi, Chae-Hyeon Sim, Ni-Na Yoo, Jin-Gyun Eom, Kyoung-Yong Jung, Eun-Ok Bang, Yoon-Seok Chung

**Affiliations:** 1 Division of Infectious Disease Research, Health and Environment Research Institute of Sejong, Sejong 30015, Republic of Korea; kskim1997@korea.kr (K.-S.K.); wls0194@hanmail.net (H.-J.J.); seoyeon1203@korea.kr (S.-Y.K.); inhae@korea.kr (I.-H.C.); stellachsim@korea.kr (C.-H.S.); fkdhs78@korea.kr (N.-N.Y.); jaguar534@korea.kr (J.-G.E.); jungky71@korea.kr (K.-Y.J.); 2 Division of High-Risk Pathogens, Bureau of Infectious Disease Diagnosis Control, Republic of Korea Disease Control and Prevention Agency (KDCA), Cheongju 28159, Republic of Korea; isi0717@korea.kr

**Keywords:** enteroviruses, daycare center, environmental hygiene management, droplet digital PCR, surface contamination, virus transmission

## Abstract

Enteroviruses (EVs) are major pathogens transmitted via direct and indirect contact, with children being particularly susceptible. As EVs persist on surfaces, environmental hygiene is critical in communal environments. We investigated EVs presence on environmental surfaces in daycare centers from April to July 2024. Environmental samples (300) were collected from floors, toys, and desks. Viral RNA was extracted and analyzed using real-time reverse transcription polymerase chain reaction (real-time RT-PCR) and ddPCR to detect pan-Enterovirus (pan-EVs) and Enterovirus D68 (EV-D68). EVs were detected in 45.3% of the samples. The detection rate refers to the combined results, including both ddPCR and real-time PCR. Specifically, pan-EVs were found in 88 samples (1.12–505 copies/20 μL) and EV-D68 in 104 samples (1.12–309 copies/20 μL). Floors (31%) were the most contaminated surfaces. Monthly analysis showed a gradual decrease in detection rates from 88.6% in April to 18.5% in July, appearing to align with the implementation of enhanced hygiene measures. However, this trend may also reflect multifaceted factors, including natural viral reduction, exclusion of symptomatic children, and increased hygiene awareness. Notably ddPCR (83.0%) exhibited nearly twice the detection rate of real-time RT-PCR (42.5%), identifying low-level viral persistence. These findings suggest that environmental surfaces serve as reservoirs for transmission, and integrating sensitive detection like ddPCR with proactive hygiene management may help mitigate EVs spread.

## 1. Introduction

Viruses, transmitted via various routes, account for approximately 60% of all human infections worldwide [1]. They are a major cause of childhood infections, spreading not only through direct contact but also through indirect contact with contaminated objects [2]. EVs are prevalent mainly in temperate regions during the summer and early autumn, and Sejong is similarly situated within a temperate climate zone. Although most infections are asymptomatic, EVs can cause neurological disorders and other illnesses. These viruses can survive indoors for several days, posing a transmission risk from asymptomatic individuals [3,4,5].

EV-D68 was first identified in 1962 and was rarely reported until the 2014 outbreak in the United States, which continued until early 2015. This virus primarily affects children and can cause a wide range of respiratory symptoms, ranging from mild illness to severe respiratory distress [6].

Keswick et al. [7] suggested that asymptomatic viral shedding via infected individuals in daycare centers can serve as a source of infection for uninfected children and their families. Some EVs can survive on fomites for extended periods, acting as secondary sources of infection. Hand washing, strict hygiene management, and proper disinfection measures have been emphasized in high-risk environments, such as daycare centers, hospitals, and restaurants [8].

EVs are non-enveloped viruses that can persist on fomites for weeks to months [9]. Studies have shown that echoviruses, coxsackieviruses, and polioviruses can remain infectious for 2–12 d or longer on painted wood, glass, and fabric surfaces [10]. Existing research indicates that viral surface persistence plays a crucial role in the spread of infection [1], highlighting the importance of understanding environmental transmission routes [11]. Epidemiological studies have confirmed that contaminated surfaces are potential vectors for disease transmission [9].

Pappas et al. [12] detected rhinovirus and EVs RNA in approximately 20% of objects in pediatric waiting rooms, suggesting that these objects can act as transmission vectors. Additionally, EVs can survive on nonporous surfaces (e.g., countertops) for at least 45 d; even small amounts of the virus can cause infections [9]. Many viral infections in healthy adults are asymptomatic or clinically inconspicuous, which complicates transmission control. Fomites, including both porous and nonporous surfaces that harbor pathogens, play a critical role in the spread of infections [9].

The droplet digital polymerase chain reaction (ddPCR) method allows for accurate detection and quantification of low target substance concentrations in environmental samples by separating the sample into tens of thousands of tiny droplets and performing independent PCR reactions in each droplet. Moreover, as ddPCR reactions occur in isolated droplets, they are less affected by various inhibitory substances, such as soil, organic matter, and chemicals, in environmental samples compared to real-time PCR. Therefore, ddPCR is highly reliable for the analysis of environmental samples [13,14]. Based on ddPCR, investigating the presence of trace amounts of viruses in the environment of daycare centers—such as on floors, toys, and other surfaces—while being minimally affected by various contaminants like soil, dust, and detergent residues, is a highly appropriate approach.

In this study, we aimed to investigate EVs in daycare center environments using sensitive ddPCR and examine the dynamics of viral presence in relation to environmental cleaning and disinfection. Through an observational field study, we evaluated hygiene management by confirming the presence of EVs on frequently touched surfaces. Consequently, this research underscores the importance of public health surveillance, with a primary focus on monitoring viral trends within real-world settings.

## 2. Materials and Methods

### 2.1. Environmental Sample Collection

From April to July 2024, 300 environmental samples were collected at two-week intervals over seven sampling sessions from a daycare center in Sejong. Samples were collected in a viral transport medium (ASAN Transport Medium(IS2) Blister, AM608-26) (ASAN Pharmaceutical, Seoul, Republic of Korea) and transported under refrigerated conditions (2–8 °C).

The samples were collected from all representative functional areas within the daycare center, covering 12 locations such as classrooms, playrooms, hallways, and dining rooms used by children aged 0–5 years. Rather than employing random sampling, we utilized a risk-based targeted sampling strategy focusing on high-touch objects and surfaces, including toys, books, desks, and mats. The selection of these surfaces was based on preliminary observations of children’s daily activity patterns and contact frequencies, identifying them as primary ‘fomites’ for potential transmission. This approach was specifically chosen as it is more suitable for assessing actual transmission risks by capturing viral presence at key points of frequent human-to-surface interaction under real-world conditions. To maintain consistency across the longitudinal study, sampling was conducted during active hours when human-to-surface interactions were most frequent, ensuring that the detected viral presence reflected the maximum potential exposure risk. Surfaces were swabbed over a 10 × 10 cm area using a single swab [15]. By conducting longitudinal sampling across seven events, we aimed to capture the fluctuating environmental viral load and provide a more representative profile of the daycare center’s overall viral presence. For large surfaces (e.g., floors, mat), up to four swabs were used for collection, and the samples were pooled for analysis. After cleaning or disinfection, to check for a reduction in virus detection, the same surfaces, such as stair railings and floors, were repeatedly sampled. The number of samples collected from each target was as follows: 82 from floors, 52 from toys, 36 from desks, 28 from mats, 16 from shelves, 10 from teaching aids, 8 from books/bookcases, 14 from chairs, and 54 from other sources such as carts and handles.

### 2.2. RNA Extraction, Real-Time RT-PCR, and ddPCR Analysis

Samples were transported in 2 mL of viral transport medium (VTM), and 280 μL was taken from it. A total of 60 μL of RNA was extracted using the QIAamp Viral RNA Mini QIAcube Kit (Qiagen, Hilden, Germany) and QIAcube Connect (Qiagen, Hilden, Germany). To detect EVs in the environment, the 7500 Fast Real-Time RT-PCR (Applied Biosystems, Foster City, CA, USA) and droplet digital PCR (Bio-Rad, Hercules, CA, USA) instruments were used. Real-time RT-PCR was performed using a human EV/EV71 multiplex real-time PCR kit (Cat. R0221) (Kogenbiotech, Seoul, Republic of Korea), which is a widely used method for EVs detection.

To overcome the challenge of extremely low viral loads in environmental samples, a two-step ddPCR assay was implemented. This strategy focuses on enhancing the detection sensitivity to monitor viral presence and its longitudinal changes following cleaning or disinfection, rather than assessing infectivity. In the first step, target-specific pre-amplification was performed using the PreAmplification pool (REF 4441856) and TaqPath 1-step Master mix (REF A15299) on the ProFlex PCR System (all from Applied Biosystems, Foster City, CA, USA).

To maximize detection sensitivity while mitigating potential quantitative bias, the pre-amplification was set at 14 cycles. This cycle number was selected in accordance with the manufacturer’s recommendations (10~14 cycles, TaqMan PreAmp Master Mix, Applied Biosystems) and by referring to previous validation studies on low-abundance targets using ddPCR [16]. This ensures that the reaction remains within the exponential phase to avoid potential amplification bias.

In the second step, the pre-amplified products were analyzed using a Custom Multiplex assay (REF CCU002) (Applied Biosystems, Foster City, CA, USA) and 2x ddPCR Supermix for Probes (No dUTP; REF 1863023) (Bio-Rad, Hercules, CA, USA).

The assays included pan-Enteroviruses (pan-EVs) and Enterovirus D68 (EV-D68). The use of standardized commercial assays with specific Assay IDs (pan-EVs: Vi06439631_s1; EV-D68: Vi06439669_s1) was prioritized to ensure inter-laboratory reproducibility and validated specificity. The pan-EVs assay consists of 86 target strains, while the EV-D68 assay includes 2 target strains, with the EV-D strain overlapping in both assays. A custom control (Applied Biosystems, Foster City, CA, USA) was used as a positive control for gating in ddPCR, with each positive droplet distinguished by color. To ensure clarity in distinguishing between the different analytical methods, enteroviruses detected by the human EV/EV71 multiplex real-time PCR kit are specifically referred to as ‘EV’, while those detected by ddPCR are referred to as ‘pan-EVs’ and ‘EV-D68’ throughout this study.

### 2.3. Real-Time RT-PCR and Droplet Digital PCR Conditions

Real-time RT-PCR was performed using a standardized protocol provided by the Human EV/EV71 Multiplex Real-Time PCR Kit (Kogenbiotech, Seoul, Republic of Korea). The real-time RT-PCR kit contains 15 µL of primer/probe per well, and only 5 µL of RNA needs to be added per well (The positive cutoff Ct value for EV: ≤33.4, EV71: ≤36.1). For ddPCR, custom conditions were set to simultaneously detect pan-EVs and EV-D68. For quantitative analysis, the first PCR reaction was prepared with a total reaction volume of 10 μL using 2.5 μL of the PreAmplification Pool, 2.5 μL of TaqPath 1-Step Master Mix, and 5 μL of extracted RNA. The amplification was performed under the conditions listed in Table 1. After amplification, the DNA concentration was measured using a Nanodrop spectrophotometer and diluted to approximately 330 ng.

For the second amplification, a mixture was prepared with 11 μL of 2× ddPCR Supermix for Probes (No dUTP), 2 μL of Custom Multiplex Assay (900 nM), χ μL of the first PCR product, and DW (1 − χ) μL to achieve a final volume of 22 μL. The second amplification step was performed under the conditions listed in Table 2.

### 2.4. Statistical Analysis

The detection rates of enteroviruses were analyzed based on fomites, monthly case counts, testing methods, and the proportion of national patients. Statistical analysis and graphical representation were performed using Microsoft Excel (Microsoft Corp., Redmond, WA, USA).

## 3. Results

### 3.1. Detection of EVs via Real-Time RT-PCR and ddPCR

**Figure 1:**
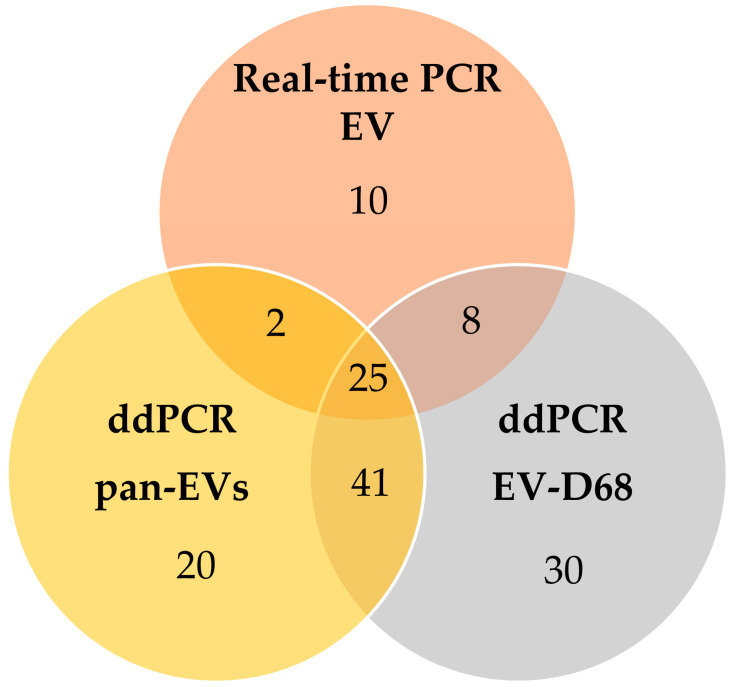
Venn diagram illustrating the overlap of detected samples among assays.

Of the 300 environmental samples, a total of 136 (45.3%) tested positive for EVs by at least Real-time RT-PCR or ddPCR. Among these, 45 samples were positive real-time RT-PCR (Ct range: 27.50–36.98), while EV71 was not detected in any sample. Quantitative ddPCR analysis identified 88 samples positive for pan-EVs (range: 1.12–505 copies/20 µL) and 104 samples for EV-D68 (range: 1.12–309 copies/20 µL). Overlapping detection between Real-time RT-PCR (for EV) and ddPCR (for pan-EVs) was observed 27 samples. Furthermore, 66 samples were positive for pan-EVs and EV-D68 within the ddPCR assays. A total of 25 samples showed overlapping positive results across all three detection targets: Real-time RT-PCR EV, ddPCR pan-EVs, and ddPCR EV-D68 (Figure 1). Notably, no significant positive correlation was observed between the Ct values of Real-time RT-PCR and the copy numbers obtained from ddPCR.

### 3.2. Distribution and Temporal Trends of Viral Detection

**Figure 2:**
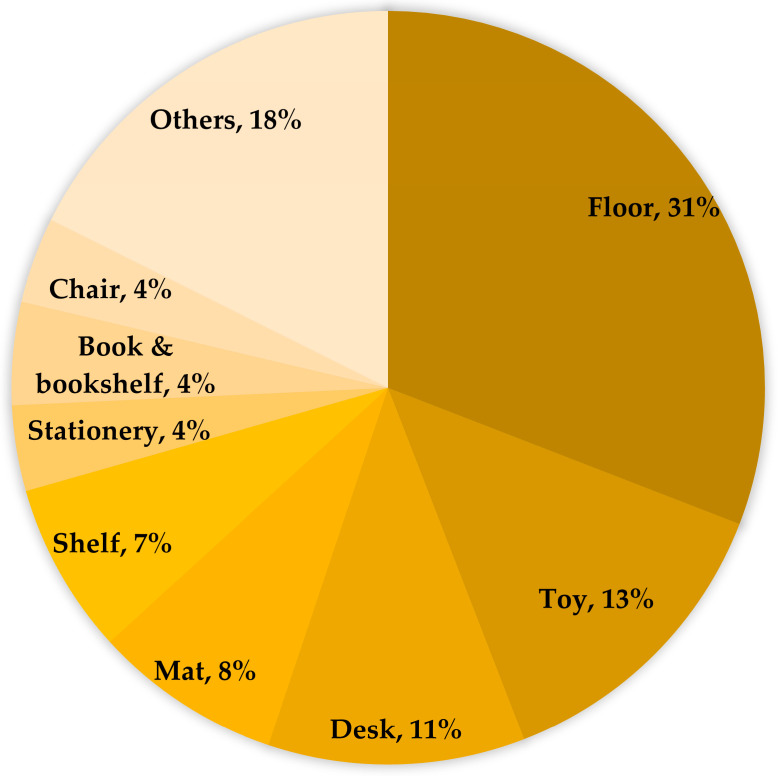
Detection rate of Enteroviruses (EVs) based on fomites.

Among the sampled surfaces, viral detection was primarily distributed across three categories—floors, toys, and desks—which collectively accounted for more than half (55%) of the total instances. Within this group, the floor represented nearly one-third (31%) of the entire distribution, a proportion exceeding the frequency found on toys and desks by more than twofold. Additionally, other surfaces including stair handrails, door handles, cart handles, elevator control panels, and child-riding carts comprised nearly one-fifth (18%) of the total. These data illustrate the viral distribution observed across the targeted surfaces, ranging from expansive floor areas to specific high-contact objects (Figure 2).

**Figure 3:**
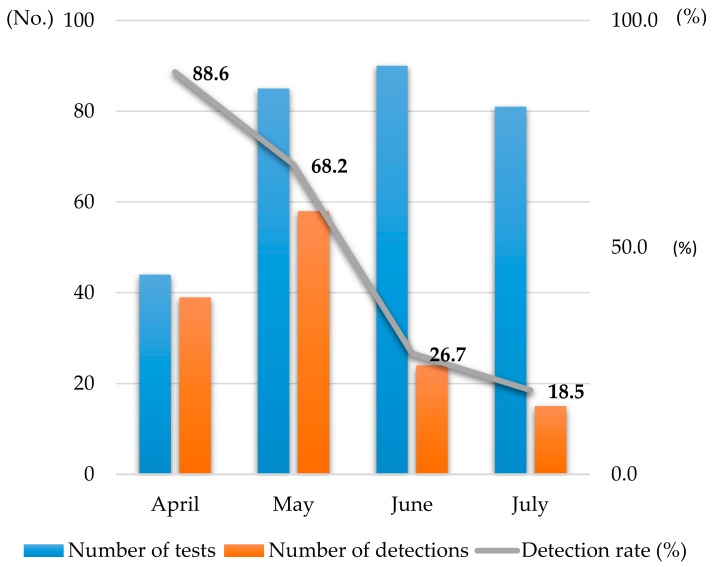
Enteroviruses(EVs) detection rate based on the monthly sample count.

Throughout the study period, a downward trend in EVs detection was observed across seven sampling sessions. The detection rate peaked in April at 88.6% and underwent a sustained reduction, reaching its minimum of 18.5% in July. These data illustrate the temporal trend of viral detection across the sampled surfaces (Figure 3).

**Figure 4:**
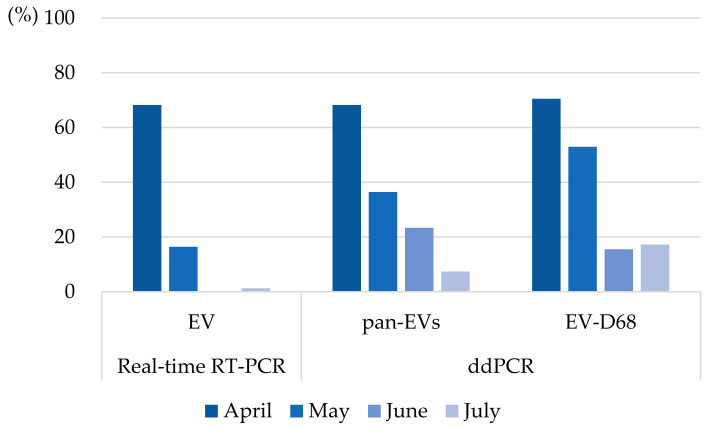
Monthly Enterovirus detection rate based on the testing method.

Consistent with the general temporal trend, both real-time RT-PCR and ddPCR methodologies exhibited synchronized monthly trajectories. While absolute detection rates were higher in ddPCR, both platforms recorded an overall decline in viral detection for EV, pan-EVs, and EV-D68 from April to July. These results suggest that viral RNA levels across the sampled surfaces followed a largely downward trend throughout the study period, regardless of the detection method used (Figure 4).

### 3.3. Comparison of Environmental Viral Loads with Nationwide Outbreak Trends

**Figure 5:**
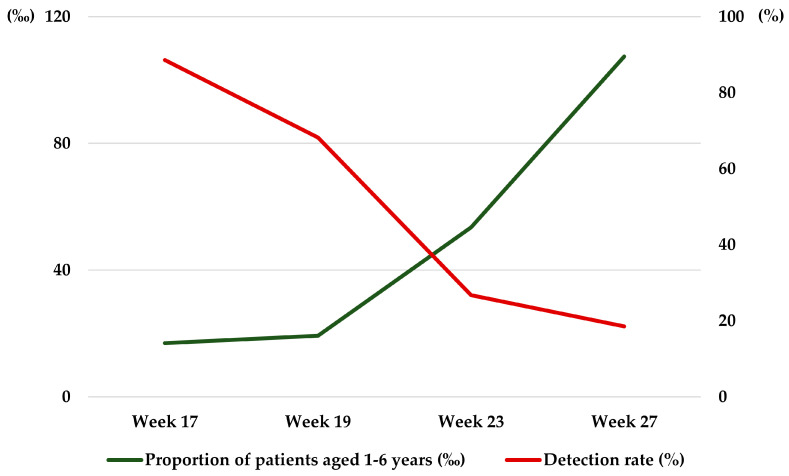
Comparison of environmental EVs detection rates in the study facility with nationwide HFMD patient proportions (Weeks 17–27). Patient proportion (‰) = (Number of HFMD cases/Total number of outpatients at sentinel surveillance sites) × 1000. Data were obtained from the KDCA Infectious Disease Surveillance System, where HFMD cases include patients diagnosed based on clinical symptoms such as fever and vesicular rashes on the hands, feet, and mouth.

The temporal trend of EVs detection in the daycare center was compared with nationwide Hand, Foot and Mouth Disease (HFMD) patient proportion data from the Korea Disease Control and Prevention Agency (KDCA) Infectious Disease Web Portal [https://dportal.kdca.go.kr/pot/is/st/hfmd.do (accessed on 19 February 2025)] between weeks 17 and 27. While the nationwide patient proportion showed a continuous increase toward a peak during this period, the environmental EVs detection rate in the study facility exhibited a contrasting downward trend (Figure 5).

Detailed follow-up testing was conducted at week 19 on specific locations (e.g., stair handrails and floors) that had previously tested positive in week 17. The results showed that both EV (via Real-time RT-PCR) and pan-EVs (via ddPCR) were no longer detectable at these sites. Furthermore, EV-D68 was either not detected or showed a significant reduction in copy numbers (Table 3).

When comparing the two analytical methods, EV were detected in 45 samples by Real-time RT-PCR, whereas pan-EVs were detected in 88 samples by ddPCR, indicating that ddPCR achieved an approximately twofold higher detection rate in these environmental samples.

## 4. Discussion

A previous study reported that infectious EVs persisted on all tested surfaces, with the longest persistence occurring at 4 °C. There was no significant difference in viral stability between porous and nonporous toys. Smooth, nonporous surfaces have also been found to be more supportive of viral stability [17]. Similarly, in our study, desks, shelves, bookcases, and chairs were made of wood, but desks had a smooth surface because they were covered with vinyl acetate, which may have influenced viral stability. Consequently, a relatively higher virus detection rate may have been observed on desks. Additionally, floors composed of harder and smoother materials than mats likely provided favorable conditions for virus persistence, resulting in higher detection rates. In contrast, EVs on toys were primarily detected on non-rigid materials, such as cloth and wooden blocks, rather than on plastic. Other samples included infant carts, carts, stairs, and door handles, elevator interiors, elevator buttons, food trays, and forks, with handles, elevator interiors, and cart interiors showing the highest detection rates.

EVs can survive on fomites for extended periods; factors such as surface type, temperature, and humidity can affect their persistence [8]. The persistence and stability of viruses are influenced by various factors, including environmental surface materials, temperature, humidity, and the concentration and type of the virus [17]. Consequently, viral persistence on surfaces is affected not only by internal factors, such as the characteristics of the surface and virus, but also by external factors, such as temperature and humidity. Viruses can cause infections, even in small amounts, if they survive on surfaces long enough to come into contact with the host. Several studies have shown that most respiratory and enteric viruses can survive in fomites and hands under certain conditions and can be transferred from fomites to hands. Therefore, disinfecting both the fomites and hands is an important method for blocking viral transmission [9]. Particularly, as EVs can survive on various surfaces, close contact among children in crowded classrooms and placing contaminated toys or utensils into their mouths pose a high risk of exposure. Other studies have suggested that using appropriate disinfectants and regular disinfection can more rapidly inactivate EVs [17].

This study had limitations in comparing virus detection rates based on surface type because it did not account for the variations in viral survival curves across different materials [18] or that environmental variability, such as high humidity, affects EVs persistence [19], or sampling scope and absence of infectivity assays. Although some studies suggest a potential discrepancy between viral RNA detection and viral infectivity [2], analysis using ddPCR is highly useful for accurate and sensitive detection of minute amounts of viruses in environmental samples compared to Real-time RT-PCR, indicating that ddPCR can more accurately measure viral load, making it a crucial tool for environmental surveillance and preventing virus spread. Therefore, ddPCR may play an important role in the prevention and control of infectious diseases in public places and work environments.

The World Health Organization (WHO) emphasizes the importance of hygiene habits for maintaining health, preventing disease spread, and stresses the need to simultaneously promote hand hygiene and environmental disinfection to improve public health [19,20]. Hand hygiene and environmental disinfection are crucial for preventing infectious diseases [20,21,22]. A key finding of this study is the marked decline in environmental viral detection rates—from 88.6% in April to 18.5% in July—which directly contrasts with the nationwide increase in HFMD cases during the same period (Figure 5). This divergence suggests that the decrease in viral presence was not merely a reflection of community trends but was likely driven by a combination of localized factors. Specifically, this downward trend can be attributed to the interplay of three synergistic factors. First, the administrative exclusion of symptomatic children served as a primary barrier, reducing the introduction of new viral loads. Second the heightened hygiene awareness prompted by the regular investigation visits likely resulted in improved adherence to environmental cleaning and ventilation practices. Finally, the implementation of targeted disinfection using 70% alcohol and 0.1% sodium hypochlorite likely contributed to the inactivation of viruses, as evidenced by the disappearance of EVs at previously positive sites within two weeks (Table 3).

The application of ddPCR for detecting trace amounts of viruses in daycare environments was an advanced approach. However, there are certain limitations to this study. As this research was conducted in an active daycare center rather than a controlled setting, it was challenging to strictly document all environmental conditions or account for the precise rate of natural viral reduction. Despite these constraints, the downward trend in viral detection appeared to align with the period when enhanced environmental hygiene measures were implemented. While these findings do not definitively establish a causal relationship, they point toward a potential relationship between intensified environmental management and the reduction in viral loads on surfaces. This implies that environmental hygiene management, alongside other factors, may have a role in mitigating the presence of viruses in communal settings.

## 5. Conclusions

This study investigated the presence of EVs in the indoor environment of daycare centers using real-time RT-PCR and ddPCR. EVs were detected through both methods, with ddPCR identifying more than twice as many positive samples as real-time RT-PCR, demonstrating its superior sensitivity for detecting trace viral loads in environmental settings. Our findings suggest that environmental surfaces may serve as potential reservoirs for viral transmission in communal spaces.

Notably, the significant reduction in viral detection observed two weeks after the initial sampling underscores the impact of proactive management. While it is challenging to isolate a single cause, this decline likely reflects the synergistic effect of targeted disinfection, heightened hygiene awareness among staff, and the administrative exclusion of symptomatic children. These results highlight the critical role of sustained environmental hygiene management in mitigating viral presence. Although this study did not assess virus infectivity or viability, our findings provide a foundation for future research to incorporate these aspects. In conclusion, establishing systematic environmental management and sensitive surveillance tools like ddPCR is essential for protecting infection-vulnerable populations in daycare settings.

## Figures and Tables

**Table 1:** One-step thermal cycling conditions.

Temperature	Time	Cycle
25 °C	2 min	1
50 °C	30 min	
95 °C	2 min	
95 °C	15 s	14
55 °C	2 min	
99.9 °C	10 min	1

**Table 2:** Two-step thermal cycling conditions.

Temperature	Time	Cycle
95 °C	10 min	1
94 °C	30 s	40
60 °C	1 min	
98 °C	10 min	1

**Table 3:** Comparison before and after disinfection and cleaning.

Description	Real-Time RT-PCR		ddPCR			
	EV (Ct Value)		Pan-EVs (Copies/20 ㎕)		EV-D68 (Copies/20 ㎕)	
	Week 17	Week 19	Week 17	Week 19	Week 17	Week 19
Stair handrail	34.31	Undetected	Undetected	Undetected	13.2	Undetected
Floor (1-year-old classroom)	29.87	Undetected	470	Undetected	235	30.2
Floor (5-year-old classroom)	33.14	Undetected	13.1	Undetected	26.3	13.1
Floor (playroom)	29.31	Undetected	128	Undetected	28.5	Undetected

## Data Availability

The raw data supporting the conclusions of this article will be made available by the authors on request.
